# Complete mitochondrial genome of the orange-spotted trevally, *Carangoides bajad* (Perciformes, Carangidae) and a comparative analysis with other Carangidae species

**DOI:** 10.1080/23802359.2020.1797587

**Published:** 2020-08-03

**Authors:** Ha Yeun Song, Yun-Hwan Jung, Young Ji Choi, Bora Kim, Tu Van Nguyen, Dae-Sung Lee

**Affiliations:** a Department of Genetic Resources Research, National Marine Biodiversity Institute of Korea, Janghang-eup, Republic of Korea; b International Center for Marine Biodiversity, National Marine Biodiversity Institute of Korea, Janghang-eup, Republic of Korea; c Department of Ecology, Institute of Tropical Biology, Vietnam Academy of Science and Technology, Ho Chi Minh City, Vietnam

**Keywords:** Mitochondrial genome, Perciformes, Carangidae, *Carangoides bajad*

## Abstract

The complete mitochondrial genome of the orange-spotted trevally, *Carangoides bajad*, which belongs to the family Carangidae was determined. The complete mitochondrial genome has a length of 16,556 bp and consists of 13 protein-coding genes, 22 tRNA genes, two rRNA genes, and a control region. *Carangoides bajad* has a mitochondrial gene arrangement that is typical of vertebrates. Phylogenetic analysis using mitochondrial genomes of 13 related species revealed that *C. bajad* formed a well-supported monophyletic group with the other Carangidae species.

The orange-spotted trevally, *Carangoides bajad*, (Perciformes, Carangidae), is an inshore marine fish that inhabits the Indo-Wet Pacific (Smith-Vaniz 1984). Carangids are an important food source in the commercial fisheries industry in Southeast Asia (Mohsin and Ambak 1996). The *C*. *bajad* is listed as Least Concern in IUCN Red List due to fishing and harvesting aquatic resources (Smith-Vaniz and Williams 2016). In this study, we determined the complete mitochondrial genome sequence of *C. bajad* and compared the sequence with those of other species of Carangidae.

The *C. bajad* specimen was collected from Ho Chi Minh City, Vietnam (10.53 N, 106.45 W). Total genomic DNA was extracted from the specimen tissue, which has been deposited at the National Marine Biodiversity Institute of Korea (Voucher No. MABIK0002416). The mitogenome was sequenced using Illumina Hiseq 4000 sequencing platform (Illumina, San Diego, CA) and assembled with *SOAPdenovo* at Macrogen Inc. (Korea). The complete mitochondrial genome was annotated using MacClade ver. 4.08 (http://macclade.org/macclade) (Maddison and Maddison 2005) and tRNAscan-SE ver. 2.0 (http://lowelab.ucsc.edu/tRNAscan-SE) (Lowe and Chan 2016).

The complete mitochondrial genome of *C. bajad* (GenBank accession no. LC557137) is 16,556 bp in length and includes 13 protein-coding genes, 22 tRNA genes, 2 rRNA genes, and a control region. The overall base composition is 28.35% A, 29.77% C, 15.83% G, and 26.05% T. All tRNA genes can fold into a typical cloverleaf structure, with lengths ranging from 67 to 75 bp. The *12S rRNA* (950 bp) and *16S rRNA* genes (1719 bp) are located between tRNA^Phe^ and tRNA^Val^ and between tRNA^Val^ and tRNA^Leu(UUR)^, respectively. Of the 13 protein-coding genes, 12 start with ATG; the exception being *COI*, which starts with GTG. The stop codon of the protein-coding genes is TAA (*ND1*, *COI*, *ATP8*, *ND4L*, *ND5*, and *ND6*), T— (*ND2*, *COII*, *ND3*, *ND4*, and *Cytb*) and TA– (*ATP6* and *COIII*). A control region (860 bp) is located between tRNA^Pro^ and tRNA^Phe^.

The phylogenetic trees were constructed by the maximum-likelihood method with 1000 replicates using MEGA 7.0 software (MEGA, Philadelphia, PA) (Kumar et al. 2016). We compared the phylogenetic trees of the newly sequenced genome and 13 other complete Carangidae (Caranginae and Naucratinae) species mitochondrial genome sequences acquired from the National Center for Biotechnology Information. We confirmed that *C. bajad* formed a monophyletic group with the other Caranginae species (Figure 1). This mitochondrial genome could be helpful for developing a conservation strategy.

**Figure 1.**
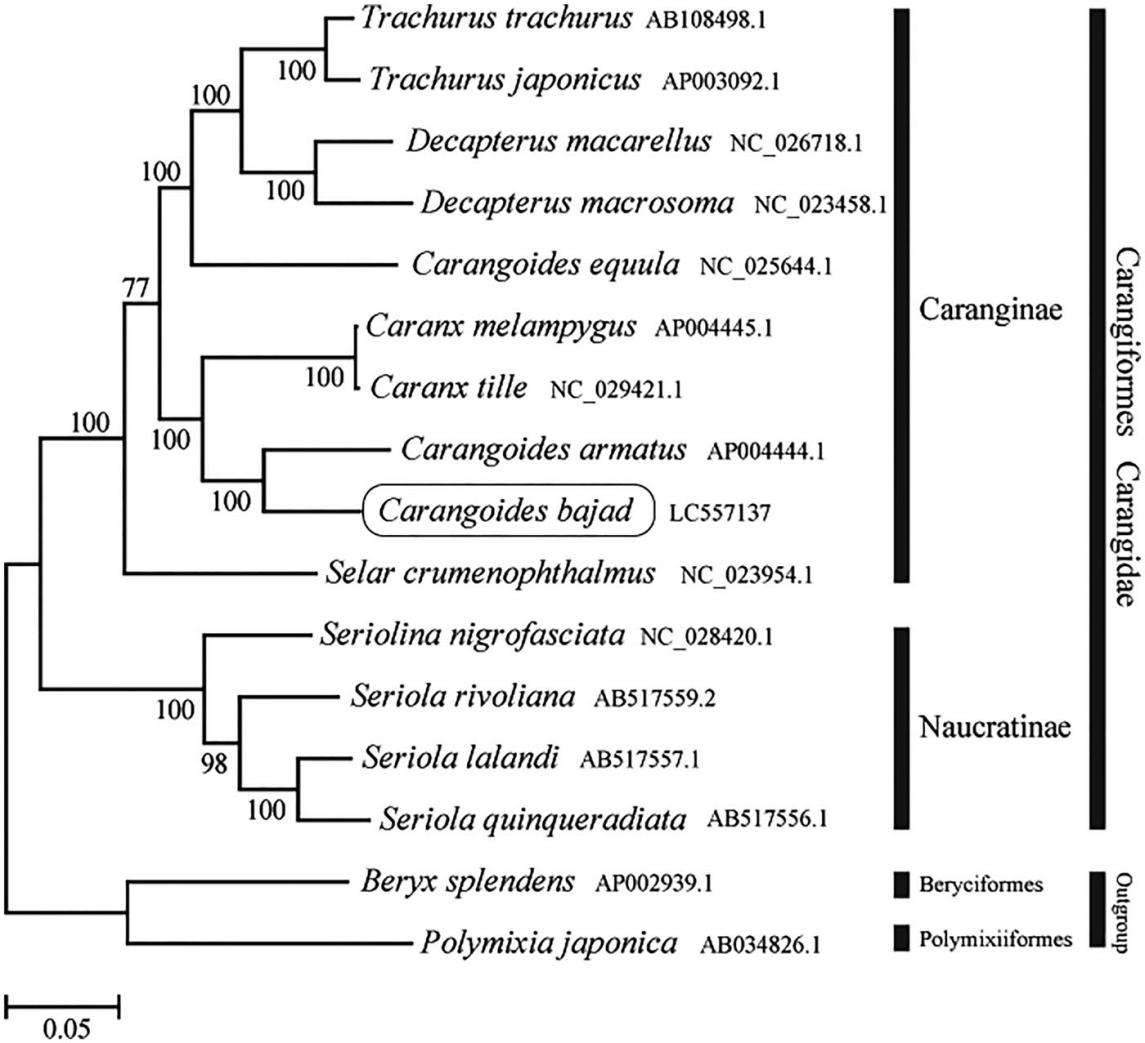
Phylogenetic position of *Carangoides bajad* based on a comparison with the complete mitochondrial genome sequences of 13 other Carangidae species. The analysis was performed using MEGA 7.0 software. The accession number for each species is indicated after the scientific name.

## Data Availability

The data that support the findings of this study are openly available in the DNA Data Bank of Japan (accession no. LC557137) at https://www.ddbj.nig.ac.jp.
